# Prevalence and Risk Factors of Active TB among Adult HIV Patients Receiving ART in Northwestern Tanzania: A Retrospective Cohort Study

**DOI:** 10.1155/2018/1346104

**Published:** 2018-07-04

**Authors:** Daniel W. Gunda, Simon C. Maganga, Igembe Nkandala, Semvua B. Kilonzo, Bonaventura C. Mpondo, Elichilia R. Shao, Samwel E. Kalluvya

**Affiliations:** ^1^Department of Medicine, Weill Bugando School of Medicine, 1464 Mwanza, Tanzania; ^2^Department of Medicine, College of Health Sciences, University of Dodoma, 395 Dodoma, Tanzania; ^3^Department of Infectious Disease, Kilimanjaro Christian Medical College, Moshi, Tanzania; ^4^Department of Medicine, Bugando Medical Centre, 1370 Mwanza, Tanzania

## Abstract

**Introduction:**

Although ART has improved the outcome of people living with HIV/AIDS, still some patients develop TB while receiving ART. The literature on the magnitude of this problem is still scarce in our setting especially northwestern Tanzania. This study was designed to determine the prevalence of active TB among HIV patients on ART and assess its potential risk factors.

**Methods:**

A retrospective cohort study was done among adult HIV-positive patients initiated on ART at Bugando Medical Centre. Patients who were TB positive before ART initiation were excluded. Data regarding demographic, clinical, and laboratory information, TB status on receipt of ART, and time on ART were collected and analyzed using STATA 11 to determine the prevalence of TB and its associated factors.

**Results:**

In total, 391 patients were enrolled in this study. The median age was 39 (32–46) years, and a total of 129 (32.99%) participants had CD4 counts <200 cells/*µ*l and 179 (45.78%) had WHO stage 3 and 4 illnesses. A total of 43 (11.0%) participants developed TB while receiving ART which was independently associated with male gender (OR = 2.9; *p*=0.007), WHO clinical stage 3 and 4 (OR = 1.4; *p*=0.029), baseline CD4 count <200 cells/*µ*l (OR = 9.1; *p* < 0.001), and having not used IPT (OR = 3.1; *p*=0.05).

**Conclusions:**

Active TB is prevalent among HIV patients while receiving ART in northwestern Tanzania which is independently associated with male gender, advanced HIV disease, and nonuse of IPT. Universal HIV testing could reduce late HIV diagnosis and hence reduce the risk of developing TB while receiving ART in our setting. Also IPT should be widely used for those who are negative for TB on screening.

## 1. Background

HIV/AIDS is a continuing health problem globally that causes considerably high morbidity and mortality especially in resource-limited countries. It has so far caused more than 35 million deaths since its discovery, and as of 2015, there were about 37 million people who were living with HIV/AIDS [1]. Sub-Saharan Africa (SSA) is the most struck region of the world which harbors about 71% of the world's burden of HIV/AIDS [2] of whom more than 1.4 million people are living in Tanzania, representing 4% of all people living with HIV/AIDS globally [3].

The HIV virus infects CD4-positive cells as its host cells in which it replicates causing progressive lysis and reduction of the number and quality of functional immune cells [4–6]. With time, the body fails to control the viral replication and immune paresis sets in, being marked by low CD4 counts with increased morbidity and mortality from opportunistic infections [7] with tuberculosis being the most common opportunistic presentation at HIV diagnosis [1].

TB/HIV is the most common coinfection which still carries high mortality and morbidity worldwide. The 2016 WHO report indicates that, in 2015, there were 10.4 million new TB cases worldwide with 11% of these cases being HIV coinfected. Additionally, there were 1.8 million deaths worldwide with 0.4 million occurring among HIV-positive patients [8]. Tuberculosis occurs as the first manifestation of HIV/AIDS in more than 50% of HIV-positive patients [9], and deaths that are linked to TB are significantly high especially in sub-Saharan Africa, where in some countries, this rate is reported to be in excess of 50% [10].

The advent of ART has generally improved the prognosis of PLHA [11, 12], as reflected by an overall reduction of HIV/AIDS-related morbidity and mortality and improved survival among PLHA [13, 14]. With use of ART, occurrence of TB has been reduced by 67–80% in most study settings [15, 16]. Even with these advantages of ART, still a significant proportion of patients on ART develop active TB with a varying prevalence rate of 2.5–30.1% in most studies [17, 18]. In our setting, the literature on the magnitude of this problem is still scarce especially in the northwestern part of Tanzania. So, the aim of this study was to determine the prevalence of active TB among HIV-positive patients while receiving ART at Bugando Medical Centre in the northwestern part of Tanzania.

## 2. Materials and Methods

This was a retrospective cohort hospital-based study which was conducted at Bugando Medical Centre (BMC), HIV Care and Treatment Centre (CTC), between August 2016 and May 2017. Bugando is a tertiary and teaching university hospital for the lake and Western zone of the United Republic of Tanzania. It is located along the shores of Lake Victoria in Mwanza city. It has a catchment population of more than 16 million people from 8 catchment regions, namely, Mara, Mwanza, Geita, Shinyanga, Simiyu, Tabora, Katavi, and Kigoma. It has a bed capacity of about 1000 and more than 1000 employees, running both inpatients' and outpatients' services. CTC activities are part and parcel of routine outpatients' activities as a referral centre. HIV patients are routinely enrolled for care and treatment services from within the hospital and some from nearby facilities. At diagnosis of HIV, patients are usually screened for TB which is done using a symptom-based tool as per national TB and leprosy (NTL) guidelines [19], which includes presence of cough ≥2 weeks, hemoptysis ≥2 weeks, excessive night sweats ≥2 weeks, fever ≥2 weeks, and noticeable weight of ≥3 kilogram over 4 weeks. Patients with one or more of these symptoms undergo further testing including sputum smear and chest radiography, and those diagnosed to have TB get started on standard treatment for TB and HIV. Currently, patients who test negative for TB screen are started on isoniazid preventive therapy (IPT) which has recently been adopted in Tanzania. On subsequent follow-up, patients are also routinely screened for TB to be able to diagnose them as early as possible to improve their outcome while receiving ART. About 400–600 patients are diagnosed with TB every year.

This study included all HIV patients who were diagnosed and initiated on ART at BMC. Patients who were diagnosed to have TB before initiation of ART were excluded from the study. A minimum sample size of 384 patients was estimated using the Kish Lisle formula (1965) for cross-sectional studies assuming 10% of HIV patients developed TB while receiving ART [20] with an allowable error of 0.03 at 95% confidence interval (CI).

A CTC database was reviewed to identify all patients who were TB negative at initiation of ART. Patients' file numbers were used to retrieve the files. From the files, data of research interest were retrieved and recorded, including age, gender, address, occupation and marital status, TB status as YES or NO, and if yes, then timing of TB in months while on ART use, WHO clinical stage, on diagnosis opportunistic infection, baseline CD4, current CD4, full blood picture, and ART regimen.

The data were double entered and cleaned using Epi Info, and data analysis was done using STATA 11 software. All continuous variables were summarized as medians with interquartile range while the categorical variables were expressed as proportions with percentages. The proportion of patients who developed TB while receiving ART was calculated and expressed as percentage, and the logistic regression model was used to determine the odds ratios and 95% CI to find out the degree of association between the outcome of interest and the potential predictors of TB while using ART. In all our calculations, factors were said to have a significant statistical association with the outcome of interest if *p* < 0.05.

### 2.1. Ethical Consideration

Ethical clearance was sought from the Faculty of Medicine and from the joint Bugando Medical Centre (BMC) and CUHAS research and ethical committee. To maintain the confidentiality, the files of the patients were handled by researchers alone, and patients' identifiers were not used in analysis.

## 3. Results

### 3.1. General Characteristics of the Study Population

**Figure 1:**
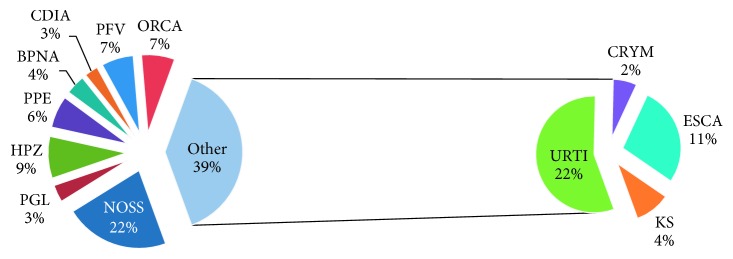
The distributions of opportunistic infection at diagnosis of HIV. BPNA: recurrent bacterial pneumonia; CDIA: chronic diarrhoea; CRYM: cryptococcal meningitis; ESCA: esophageal candidiasis; HPZ: Herpes zoster; KS: Kaposi sarcoma; NOSS: asymptomatic; ORCA: oral candidiasis; PFV: persistent fever; PGL: persistent generalized lymphadenopathy; PPE: pruritic.

**Figure 2:**
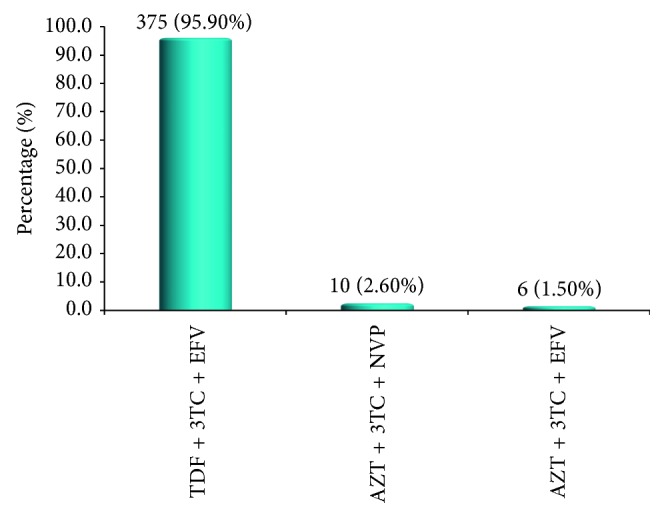
The distribution of ART regimens among 391 study participants. AZT: zidovudine; EFV: efavirenz; 3TC: lamivudine; NVP: nevirapine; TDF: tenofovir.

A total of 391 patients were enrolled in this study. The median age was 39 (32–46) years, and most patients, 295 (75.45%), were females. Of the study participants, 164 (41.94%) were married and about a third, 128 (32.74%), were doing small business. Of the studied patients, 129 (32.99%) presented with severe immune suppression and 179 (45.78%) had WHO clinical stage 3 and 4 AIDS-defining illnesses on diagnosis of HIV (Table 1) with distribution of opportunistic conditions as summarized in Figure 1. Furthermore, the majority of the studied patients, 375 (95.90%), were on tenofovir- (TDF-) based regimens, while the rest were on zidovudine- (ZVD-) based regimens (Figure 2).

**Figure 3:**
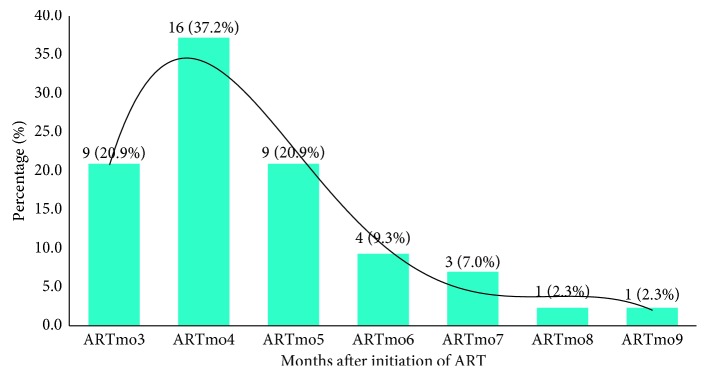
The distribution of TB occurrence by time on ART.

Of the studied patients, 43 (11.0%) developed TB while receiving ART (Table 1), whereby more than 88% of this TB occurred within the first six months of ART, as summarized in Figure 3. The odds of developing active TB while receiving ART were independently associated with male gender (OR = 2.9; *p*=0.007), having WHO clinical stage 3 and 4 AIDS-defining illness at baseline (OR = 1.4; *p*=0.029), lower CD4 count than 200 cells/*µ*l (OR = 9.1; *p* < 0.001), and having not used IPT (OR = 3.1; *p*=0.05). The difference in distribution of other factors was not statistically significant (Table 2).

## 4. Discussion

The objective of this study was to determine the prevalence of TB among adult HIV-positive patients while receiving ART and assess the associated risk factors at Bugando HIV Care and Treatment Centre. Of the 391 studied patients, 43 (11.0%) were found to develop TB while receiving ART, and the risk of developing TB was independently increased among male patients, those with WHO clinical stage 3 and 4 AIDS illnesses and CD4 <200 cells/*µ*l, and those who did not use IPT.

The prevalence rate of TB while receiving ART in the index study is similar to a previous prevalence rate of 10% reported from South Africa in 2011 [20], and it is also similar to a rate reported in 2015 from Muhimbili, where a total of 67686 were enrolled and 7602 (11.2%) were found to develop active TB while on ART follow-up program [21]. However, a much lower rate of TB was reported in 2014 from a study involving 1824 ART experienced patients in Mexico where only 45 (2.47%) developed active TB [17]. Another smaller TB rate of 5.6% was reported from the Netherlands in 2010 [22]. On the other hand, a much higher prevalence of TB was reported from a recent study in South Africa by Gupta involving 1544 patients on ART where 424 (30.1%) developed active TB in a course of 5 years [18]. The difference in TB rates could partly be explained by the overall prevalence of TB and HIV which is lower in most of the developed countries as compared to resource-restricted countries. However, even with these differences, it is important to note that though ART has significant effect on the overall occurrence of TB among HIV patients [16, 23], the occurrence of TB while receiving ART is still higher as compared to the general population as reported previously [24, 25], and it has been noted to be associated with increased mortality rate in this subgroup of patients [26, 27].

A number of factors were investigated for their potential association with the occurrence of TB while receiving ART. In the index study, male patients were found to have an increased risk of developing TB. Several studies have had similar findings including a study from South Africa by Gupta and colleagues [28], a study from Dar es Salaam Tanzania by Liu et al. [21], and also a study from Mexico by Martin-Echevarria et al. [17]. The social behavior and hormonal-related differences between male and female susceptibility to TB have been suggested as potential explanation of TB predominance among adult male patients [29, 30] as also supported by fewer studies demonstrating high prevalence of TB among female patients [31, 32]. On the other hand, gender difference in susceptibility to TB is clinically important since it has also been shown that male patients have higher risk of mortality. For instance, in a study assessing mortality in Uganda where male patients were found to be more severely ill with most of WHO clinical stage 3&4 AIDS-defining illnesses (36% versus 33%; *p* < 0.0001), they were also demonstrated to have 37% higher risk of death than female patients on receipt of ART (OR = 1.37; *p* < 0.001) [33].

Patients who had advanced HIV disease were also found to have an increased risk of developing TB while receiving ART in the index study. In Burkina Faso, patients who had lower CD4 count at diagnosis were also shown to have an increased risk of developing TB [34]. Another study from Nigeria reported similar findings that patients who had lower baseline CD4 counts <200 cells/*µ*l and those with prior history of TB had increased risk of developing TB while receiving ART [35]. Reporting 8% prevalence rate of TB while receiving ART in addition to lower CD4 counts, patients who developed TB on ART were also most likely to have a positive tuberculin skin test and prior history of admission [36]. Patients who start ART at the baseline CD4 counts <200 cells/*µ*l have frequently been demonstrated to have a subsequent poor immune recovery on receipt of ART [37, 38], taking a much long time before gaining an adequate immunity against most opportunistic infections (OIs) including TB [25]. They have also been shown to have an increased risk of both AIDS and non-AIDS-related morbidity and mortality [39, 40].

In the current study, it was also observed that 35 (81.40%) and 31 (72.09%) of those who developed TB had severe immune suppression and stage 3 and 4 AIDS-defining illness at the baseline, respectively. This is in agreement with several other studies. For instance, in 2005, Lawn and colleagues from South Africa indicated that patients who had WHO clinical stage 3 and 4 defining illnesses at the baseline were 3.6 times more likely to have TB on receipt of ART (AOR = 3.60; *p*=0.01) [24]. Also, in another study from Ethiopia by Melkamu et al., it was found that development of active TB while receiving ART was significantly higher among those patients who were in WHO clinical stage 3 and 4 as compared to those in WHO clinical stage 1 and 2 (AOR = 2.29; *p*=0.003) [41]. These findings suggest that strategies for early HIV diagnosis to increase timely diagnosis of HIV before it is adversely advanced could potentially reduce the occurrence of TB while receiving ART in our setting.

In this study also, patients who did not receive IPT while on ART were likely to develop TB as compared to those who received IPT. In 2015, one study from Ethiopia had a similar observation to our finding [42]. In this study, it was demonstrated that occurrence of TB while receiving ART was more common among those who did not use IPT as compared to those who used IPT (AHR = 2.41; *p* < 0.05). Similarly, a prior study in Dar es Salaam, Tanzania, by Liu et al. had indicated that patients who did not use IPT had increased risk of developing active TB while receiving ART (AHR = 2.25; *p* < 0.001) as compared to those who were on IPT [21]. These findings suggest that occurrence of TB while receiving ART is still a common problem in Tanzania as well and IPT could potentially reduce the TB-related morbidity and mortality in these patients. The use of IPT alone has been shown to reduce the risk of active TB by 32% among those living with HIV [43], whereas ART alone has been shown elsewhere to reduce the risk of active TB by up to 67% [15] and risk of mortality by 64–95% [44] especially when initiated timely. Concomitant use of ART and IPT was reported previously to have a much greater effect on incidence of TB of up to 80% [45].

In conclusion, these findings suggest that active TB is still a common problem among patients receiving ART in our settings. Patients who are at increased risk of developing active TB while receiving ART include male patients, those who are diagnosed with advanced HIV disease, and those who do not receive IPT. A timely diagnosis and treatment of HIV could potentially reduce the incidence of TB while receiving ART. These results also support the use of IPT among patients who are negative for TB to reduce the magnitude of this problem while receiving ART.

## Figures and Tables

**Table 1:** Distribution of demographic, clinical, and laboratory characteristics among 391 adult HIV-positive study participants.

Variables	Frequency	Percentage or median (IQR)
Age in years	391	39 (32–46)
*Gender*		
Male	096	24.55
Female	295	75.45
*Marital status*		
Single	054	13.81
Married	164	41.94
Widow	061	15.60
Divorce	112	28.64
*Occupation*		
Formal employment	025	06.39
Peasant	102	26.09
Small business	128	32.74
Driver	012	03.07
Student	009	02.30
Housewife	041	10.49
Others	074	18.93
*BMI categories*		
Under WT	062	15.86
Normal WT	225	57.54
Over WT	070	17.90
Obese	034	8.70
*WHO stage*		
3 and 4	179	45.78
1 and 2	212	54.22
*Baseline CD4 in cells/µl*		289 (125–509)
<200	129	32.99
≥200	262	67.01
*HB in g/dL*		11 (9.6–12.6)
*Anemia*		
Yes	189	48.34
No	202	51.66
*Duration on ART (mo)*		15 (11–21)
*IPT use status*		
No	103	26.34
Yes	288	73.66
*TB while on ART*		
Yes	043	11.00
No	348	89.00

ART: antiretroviral therapy; HB: hemoglobin; CD4: cluster of differentiation 4; IPT: isoniazid preventive therapy; IQR: interquartile range; mo: months; TB: tuberculosis; WHO: World Health Organization; WT: weight.

**Table 2:** Univariate and multivariate analysis for factors associated with development of active TB while receiving ART.

Variables	TB while receiving ART		Unadjusted		Adjusted	
	Yes (*N*=43)	No (*N*=348)	OR (95% CI)	*p* value	OR (95% CI)	*p* value
*Gender*						
Male	24 (55.81)	072 (20.69)				
Female	19 (44.19)	276 (79.31)	4.8 (2.5–9.3)	**<0.001**	2.9 (1.3–6.2)	**0.007**
*Age group*						
≥50 years	07 (16.28)	005 (16.67)				
<50 years	36 (83.72)	290 (83.33)	1.0 (0.4–2.2)	0.949		
*Marital status*						
Single	04 (09.30)	050 (14.37)	0.6 (0.2–1.7)	0.368		
Married	21 (48.84)	143 (41.09)	1.4 (0.7–2.5)	0.333		
Divorced	17 (39.53)	095 (27.30)	1.7 (0.9–3.3)	0.166		
Widowed	01 (02.33)	060 (17.24)	0.1 (0.0–0.8)	0.034		
*Occupation*						
Formal	02 (04.65)	023 (06.61)	0.7 (0.2–3.0)	0.622		
Peasant	06 (13.95)	096 (27.59)	0.4 (0.2–1.0)	0.061		
Petty business	18 (41.86)	110 (31.61)	1.5 (0.8–2.9)	0.179		
Driver	04 (09.30)	008 (02.30)	4.3 (1.2–15.0)	**0.027**	2.5 (0.4–13.3)	0.294
Student	02 (04.65)	007 (02.01)	2.3 (0.4–11.8)	0.290		
H/wife	05 (11.63)	036 (10.34)	1.1 (0.4–3.0)	0.796		
Others	06 (13.95)	058 (19.54)	0.7 (0.2–1.6)	0.380		
*Nutrition status*						
Under WT	14 (32.56)	048 (13.79)	3.0 (1.4–6.1)	**0.002**	1.4 (0.6–3.2)	0.463
Normal WT	21 (48.84)	204 (58.62)	0.7 (0.3–1.2)	0.223		
Over WT	06 (13.95)	064 (18.39)	0.7 (0.3–1.8)	0.476		
Obese	02 (03.23)	053 (08.76)	0.4 (0.1–2.0)	0.329		
*WHO stage*						
3 and 4	31 (72.09)	148 (42.53)				
1 and 2	12 (27.91)	200 (57.47)	3.5 (1.7–7.0)	**<0.001**	2.4 (1.0–5.2)	**0.029**
*Baseline CD4*						
<200 cells/*µ*l	35 (81.40)	094 (27.01)				
≥200 cells/*µ*l	08 (18.60)	254 (72.99)	11.8 (5.2–26.4)	**<0.001**	9.1 (3.9–21.0)	**<0.001**
*Anemia*						
Yes	22 (51.16)	167 (47.99)				
No	21 (48.84)	181 (52.01)	1.1 (0.6–2.1)	0.694		
*IPT use*						
No	39 (90.70)	249 (71.55)				
Yes	04 (09.30)	099 (28.45)	3.8 (1.3–11.1)	**0.012**	3.1 (1.0–9.4)	**0.05**
*ART regimen*						
TDF/3TC/EFV	39 (90.70)	336 (96.55)	0.3 (0.1–1.1)	0.080		
AZT/3TC/EFV	02 (04.65)	004 (01.15)	4.1 (0.7–23.6)	0.104		
AZT/3TC/NVP	02 (04.65)	008 (02.30)	2.0 (0.4–10.1)	0.367		

ART: antiretroviral therapy; AZT: zidovudine; CI: confidence interval; CD4: cluster of differentiation 4; EFV: efavirenz; 3TC: lamivudine; H/wife: housewife; IPT: isoniazid preventive therapy; NVP: nevirapine; TDF: tenofovir; WT: weight.

## Data Availability

The data used to support the findings of this study are available from the corresponding author upon request.
